# Non-hotspot PIK3CA mutations are more frequent in CLOVES than in common or combined lymphatic malformations

**DOI:** 10.1186/s13023-021-01898-y

**Published:** 2021-06-10

**Authors:** Pascal Brouillard, Matthieu J. Schlögel, Nassim Homayun Sepehr, Raphaël Helaers, Angela Queisser, Elodie Fastré, Simon Boutry, Sandra Schmitz, Philippe Clapuyt, Frank Hammer, Anne Dompmartin, Annamaria Weitz-Tuoretmaa, Jussi Laranne, Louise Pasquesoone, Catheline Vilain, Laurence M. Boon, Miikka Vikkula

**Affiliations:** 1 Human Molecular Genetics, de Duve Institute, University of Louvain, Avenue Hippocrate 74 (+5), bte B1.74.06, 1200 Brussels, Belgium; 2 Otolaryngology Department, Cliniques Universitaires Saint-Luc, University of Louvain, Brussels, Belgium; 3 Center for Vascular Anomalies, Division of Plastic Surgery, Cliniques Universitaires Saint-Luc, University of Louvain, Brussels, Belgium; 4 VASCERN VASCA European Reference Centre, Brussels, Belgium; 5 Department of Dermatology, Université de Caen Basse Normandie, CHU Caen, Caen, France; 6 Department of Otolaryngology, Kokkola Central Hospital, Kokkola, Finland; 7 Department of Otorhinolaryngology, Head and Neck Surgery, Tampere University Hospital, Tampere, Finland; 8 Service de Chirurgie Plastique Reconstructive, Hôpital Salengro, CHU de Lille, Lille, France; 9 Department of Genetics, Hôpital Erasme, ULB Center of Human Genetics, Université Libre de Bruxelles, Brussels, Belgium; 10 Walloon Excellence in Lifesciences and Biotechnology (WELBIO), University of Louvain, Brussels, Belgium

**Keywords:** Lymphatic malformation, Isolated, Gene, Mutation, Somatic, PI3K, Epidemiology, Theragnostic, Allele, Frequency

## Abstract

**Background:**

Theragnostic management, treatment according to precise pathological molecular targets, requests to unravel patients’ genotypes. We used targeted next-generation sequencing (NGS) or digital droplet polymerase chain reaction (ddPCR) to screen for somatic *PIK3CA* mutations on DNA extracted from resected lesional tissue or lymphatic endothelial cells (LECs) isolated from lesions. Our cohort (n = 143) was composed of unrelated patients suffering from a common lymphatic malformation (LM), a combined lymphatic malformation [lymphatico-venous malformation (LVM), capillaro-lymphatic malformation (CLM), capillaro-lymphatico-venous malformation (CLVM)], or a syndrome [CLVM with hypertrophy (Klippel-Trenaunay-Weber syndrome, KTS), congenital lipomatous overgrowth-vascular malformations-epidermal nevi -syndrome (CLOVES), unclassified PIK3CA-related overgrowth syndrome (PROS) or unclassified vascular (lymphatic) anomaly syndrome (UVA)].

**Results:**

We identified a somatic *PIK3CA* mutation in resected lesions of 108 out of 143 patients (75.5%). The frequency of the variant allele ranged from 0.54 to 25.33% in tissues, and up to 47% in isolated endothelial cells. We detected a statistically significant difference in the distribution of mutations between patients with common and combined LM compared to the syndromes, but not with KTS. Moreover, the variant allele frequency was higher in the syndromes.

**Conclusions:**

Most patients with an common or combined lymphatic malformation with or without overgrowth harbour a somatic *PIK3CA* mutation. However, in about a quarter of patients, no such mutation was detected, suggesting the existence of (an)other cause(s). We detected a hotspot mutation more frequently in common and combined LMs compared to syndromic cases (CLOVES and PROS). Diagnostic genotyping should thus not be limited to PIK3CA hotspot mutations. Moreover, the higher mutant allele frequency in syndromes suggests a wider distribution in patients’ tissues, facilitating detection. Clinical trials have demonstrated efficacy of Sirolimus and Alpelisib in treating patients with an LM or PROS. Genotyping might lead to an increase in efficacy, as treatments could be more targeted, and responses could vary depending on presence and type of *PIK3CA*-mutation.

**Supplementary Information:**

The online version contains supplementary material available at 10.1186/s13023-021-01898-y.

## Background

**Fig. 1:**
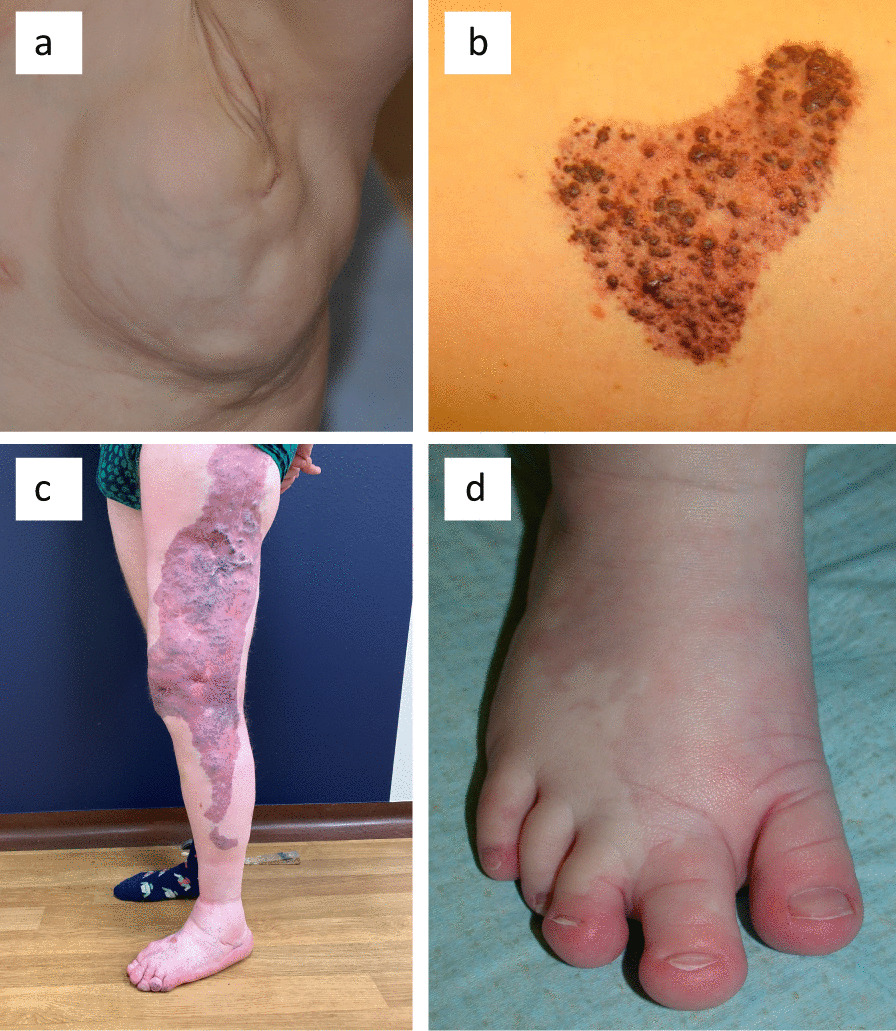
Clinical characteristics of representative patients. **a** Mixed LM of left thorax (mutation p.Glu545Lys at 5% by NGS). **b** CLVM of left thorax with visible lymphatic vesicles (mutation p.His1047Arg at 5% by NGS and 5% by ddPCR). **c** Unclassified PROS (mutation p.Cys420Arg at 2% by NGS). **d** Macrodactyly and syndactyly 2–3 with small sandal gap and CM of right foot of a CLOVES syndrome patient

Vascular malformations are usually congenital and localized. They slowly develop with the growth of the child. Treatments are mostly limited to laser, sclerotherapy, embolization and surgical resection. A malformation can affect any part of the lympho-vascular system (Fig. 1). A pure malformation affects only one compartment, e.g. a common lymphatic malformation (LM), whereas combined vascular malformations associate at least two components in the same lesion, e.g. a capillaro-lymphatic malformation [1]. Patients with isolated lesions have no other associated signs, whereas syndromic-forms affect at least one other organ.

In the princeps study in 2009, it was demonstrated that somatic genetic mutations are associated with sporadically occurring vascular anomalies: isolated venous malformations (VM) were discovered to have somatic *TIE2*/TEK mutations [2]. Since then, the etiopathogenesis of various isolated and combined vascular malformations has been unravelled [3, 4]. One of the genes identified to be mutated in vascular malformations encodes the phosphatidylinositol-4,5-bisphosphate 3-kinase catalytic subunit alpha (PIK3CA, also known as p110α). Mutations were first identified in rare syndromic patients with Congenital Lipomatous Overgrowth, Vascular malformations, Epidermal nevi, and Skeletal/spinal abnormalities and/or scoliosis (CLOVES) [5]. Subsequently, *PIK3CA* mutations were implicated in common LM (43 out of 47 lesions) [6–8], and isolated VM without a *TIE2/*TEK mutation (27 out of 50 lesions) [9]. Somatic/mosaic PIK3CA mutations have also been implicated in variable syndromic phenotypes that can associate vascular anomalies with hypertrophy, leading to name this spectrum “PIK3CA-Related Overgrowth Syndrome” or PROS [10]. It includes CLOVES, Fibroadipose Hyperplasia (FH), Fibroadipose infiltrating lipomatosis, Hemihyperplasia multiple lipomatosis (HHML), Klippel-Trenaunay syndrome (KTS), Megalencephaly-Capillary Malformation-Polymicrogyria syndrome (MCAP), Macrodactyly, Hemimegalencephaly, and Muscle hemihyperplasia [10].

**Fig. 2:**
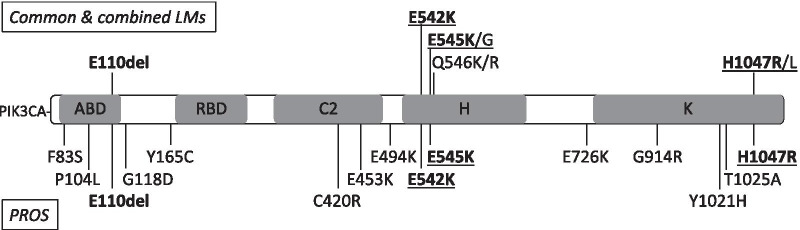
PIK3CA protein (Human, 1068 aa) and mutations. Top, mutations found in common and combined lymphatic malformations (LM, LVM, CLVM). Bottom, mutations found in patients with PROS (KTS, CLOVES, unclassified PROS). See distribution in Table 1. Shared mutations in bold. Hotspot mutations underlined. ABD, p85α-binding domain; RBD, Ras-binding domain; C2, C2-PIK3C-type domain; H, helical domain; K, kinase domain

*PIK3CA* is considered as an oncogene [11]. The encoded p110α subunit contains the adapter-binding domain (ABD), the Ras-binding domain (RBD), the C2-PI3K-type domain (C2), the helical domain (H) and the kinase domain (K) (Fig. 2) [12]. Mutations that spread over the five functional domains have been implicated in many cancers, with positions *c.1624G* (p.E542), *c.1633G* (p.E545) and *c.3140A* (p.H1047) as hotspots. Similar mutations have been found in small series of lymphatic and vascular malformations.

In this study, we analysed for the presence of a *PIK3CA* mutation in a large cohort of lymphatic malformations, including common LM, lymphatico-venous malformation (LVM), capillaro-lymphatic malformation (CLM), capillaro-lymphatico-venous malformation (CLVM), unilateral capillaro-lymphatico-venous malformation with hypertrophy (KTS), CLOVES syndrome, unclassified PROS and unclassified vascular anomaly syndrome (UVA). This study contains the largest cohort studied so far for isolated lymphatic malformations. We analysed the prevalence, distribution and allele frequency of *PIK3CA* mutations in these phenotypes and evaluated for any genotype–phenotype correlation. This study provides firm epidemiological data, essential for the design of diagnostic testing, prospective clinical trials and drug development.

## Results

To assess the contribution of PIK3CA mutations in common, combined and syndromic LM (Table 1), we screened DNA extracted from frozen tissues or isolated cells from 143 patients: 105 common LM, 3 LVM, 1 CLM, 7 CLVM, 4 KTS, 14 CLOVES, 7 PROS and 2 UVA. Within the mutated lesions, the variant allele frequency (VAF) varied strongly: from 0.54 to 25.33% for NGS-based analyses, and from 0.28% (14 SPD) to 22.7% for ddPCR-based analyses. The median VAF was 4.04% (STDEV = 4.57%), with 60% of patients having ≤ 5% VAF. In addition, we had 8 common LMs inconclusive for the presence of hotspot mutations (5 to 9 mutant reads, but only 0.24–0.52% VAF, not confirmed by ddPCR due to lack of DNA).

**Table 1:** Patients cohort and PIK3CA mutations per pathology

Pathology	LM	LVM	CLM	CLVM	KTS	CLOVES	PROS	UVA	All
Cohort	105	3	1	7	4	14	7	2	143
**Mutation**	**78**	**3**	–	**6**	**4**	**12**	**5**	–	**108**
No mutation	19	–	1	1	–	2	2	2	27
Inconclusive	8	–	–	–	–	–	–	–	8
**% with a mutation**	74.3%	100%	0%	85.7%	100%	85.7%	71.4%	0%	75.5%
VAF median	3.71	4.59	–	7.34	8.12	12.90	11.09	–	4.05
Standard deviation	4.49	2.52	–	6.53	6.27	7.30	5.65	–	4.85
VAF range	0.54–11.34	3.43–8.25	–	4.71–22.19	1.15–13.17	2.00–25.33	1.00–13.00	–	0.54–25.33
**Hotspot mutations**	**74**	**3**	**0**	**6**	**3**	**5**	**1**	**0**	**92**
Non-hotspot mutations	4	0	0	0	1	7	4	0	16

PIK3CA Domain	Mutation	LM	LVM	CLM	CLVM	KTS	CLOVES	PROS	UVA	All
ABD	F83S							1		1
ABD	P104L						1			1
	E110del	1				1				2
	G118D							1		1
	Y165C						1			1
C2	C420R							1		1
C2	E453K						1			1
	E494K							1		1
Helical	**E542K**	**26**	**3**				**1**	**1**		**31**
Helical	**E545K**	**27**			**3**	**3**	**1**			**34**
Helical	**E545G**	**1**								**1**
Helical	Q546K	2								2
Helical	Q546R	1								1
	E76K						1			1
Kinase	G914R						1			1
Kinase	Y1021H						1			1
Kinase	T1025A						1			1
Kinase	**H1047R**	**17**			**3**		**3**			**23**
Kinase	**H1047L**	**3**								**3**

Bold: hotspot mutations. Protein domains based on https://www.uniprot.org/uniprot/P42336: ABD, p85α-binding domain (amino acids 16–105); C2, C2-PIK3C-type domain (330–487); Helical, Helical domain (517–694); Kinase, Kinase domain (797–1068). See also Fig. 2

PIK3CA mutations were found in 78/105 common LM (74.3%), 3/3 LVM (100%), 6/7 CLVM (85.7%), 4/4 KTS (100%), 12/14 CLOVES (85.7%) and 5/7 PROS (71.4%) (Table 1). No *PIK3CA* mutation was detected in the unique CLM and the two UVA tested. Out of the 108 mutations detected, 92 (85.1%) were at hotspot positions: 31 p.E542K (*c.1624G* > *A*), 34 p.E545K (*c.1633G* > *A*), 1 p.E545G (*c.1634A* > *G*), 23 p.H1047R (*c.3140A* > *G*) and 3 p.H1047L (*c.3140A* > *T*). The rest was covered by 14 distinct non-hotspot mutations in 16 samples (Table 1). We also isolated primary cells from two resected tissues (one LM and one CLVM), to locate the population harbouring the mutation (Table 2). Strong enrichment of the somatic mutation was observed within the isolated endothelial cells (ECs) and especially lymphatic ECs.

**Table 2:** PIK3CA variants allele frequencies in tissues and isolated cells

Disease	Sample type	Mutation	VAF (%)
CLVM	Tissue	p.His1047Arg	5
	CD31-positive cells (EC)	p.His1047Arg	47
	CD31-negative cells	p.His1047Arg	0
LM	Tissue	p.Glu542Lys	11
	Unselected cells	p.Glu542Lys	4
	CD34-positive cells (BEC)	p.Glu542Lys	17
	CD31-positive and CD34-negative cells (LEC)	p.Glu542Lys	32
	CD31- and CD34-negative cells	p.Glu542Lys	0
	Fibroblasts	p.Glu542Lys	0

VAF, variant allele frequency; EC, endothelial cell; BEC, blood endothelial cell; LEC, lymphatic endothelial cell

**Fig. 3:**
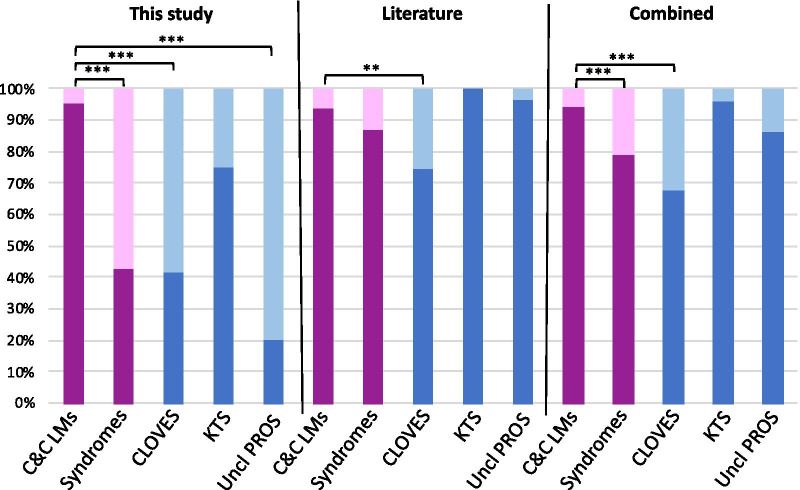
Genotype distribution of PIK3CA mutations between patients with common and combined LMs or PROS. Distribution of hotspot mutations (dark colours, affecting amino acid positions 542, 545 or 1047) and non-hotspot mutations (light colours, all other amino acid positions) in patient cohorts. C&C LMs (Common and combined LMs) includes LM, LVM and CLVM. PROS, includes CLOVES, KTS and unclassified (Uncl) PROS (shown separately in blue). Literature: from references [6–8, 10, 13, 14]. Combined: this study and the literature. See Table 3 for values. ***p value < 0.001. **p value < 0.01

We detected a statistically significant difference in the distribution of hotspot mutations and non-hotspot mutations between common and combined LM compared to the syndromes (*p* value = 1.329 × 10^–7^), to CLOVES (*p* value = 1.441 × 10^–5^) or to unclassified PROS (*p* value = 1.207 × 10^–4^), but not to KTS (Fig. 3 and Table 3). Our meta-analysis of data collected from published reports detected a similar but weaker difference for CLOVES (*p* value = 0.0023) [6–8, 10, 13, 14]. When combining our data with the literature, the mutation distribution difference was significant between common and combined LMs versus PROS (*p* value = 7.742 × 10^–5^) and versus CLOVES (*p* value = 5.575 × 10^–7^). All *p* values adjusted for multiple testing remained significant (Table 3). As earlier reports focussed on hotspot screenings, it probably explains the weaker association in the literature data. Out of the altogether 367 patients reported by us and others, 54 (14.7%) are PIK3CA-negative: 38 LM, 1 CLM, 3 CLVM, 1 KTS, 4 CLOVES, 5 PROS and 2 UVA. Some of these could just be under the threshold of detection.

**Table 3:** Comparison of the mutation types between the different cohorts

	Number of patients	Pathology	Hotspot	Non-hotspot	Fisher's Exact Test	*p* value	Adjusted *p* value	Significance
This study	n = 87	C&C LMs	83	4				
	n = 21	PROS	9	12	C&C LMs vs PROS	1.329e-07	1.5948e-06	***
	*12*	*CLOVES*	*5*	*7*	*C&C LMs vs CLOVES*	*1.441e-05*	*5.764e-05*	***
	*4*	*KTS*	*3*	*1*	*C&C LMs vs KTS*	*0.2055*	*0.274*	
	*5*	*Uncl PROS*	*1*	*4*	*C&C LMs vs Uncl PROS*	*0.0001207*	*0.0002896*	***
Literature	n = 107	C&C LMs	100	7				
	n = 98	PROS	85	13	C&C LMs vs PROS	0.1562	0.2343	
	*47*	*CLOVES*	*35*	*12*	*C&C LMs vs CLOVES*	*0.002331*	*0.004662*	**
	*20*	*KTS*	*20*	*0*	*C&C LMs vs KTS*	*0.5955*	*0.7146*	
	*31*	*Uncl PROS*	*30*	*1*	*C&C LMs vs Uncl PROS*	*0.6832*	*0.7453*	
Combined	n = 194	C&C LMs	183	11				
	n = 119	PROS	94	25	C&C LMs vs PROS	7.742e-05	2.232e-04	***
	*59*	*CLOVES*	*40*	*19*	*C&C LMs vs CLOVES*	*5.575e-07*	*3.345e-06*	***
	*24*	*KTS*	*23*	*1*	*C&C LMs vs KTS*	*1*	*1*	
	*36*	*Uncl PROS*	*31*	*5*	*C&C LMs vs Uncl PROS*	*0.1434*	*0.2343*	

C&C (common and combined) LMs: LM, LVM, CLVM. PROS: includes CLOVES, KTS and unclassified (Uncl) PROS. Detailed values of PROS subgroups are given in italic. Literature: from references [6–8, 10, 13, 14]. Combined: this study and the literature. **p value < 0.01; ***p value < 0.001. The distributions between hotspot and non-hotspot are shown in Fig. 3

We also studied variability of VAF in different samples of the same patient. We had 16 patients for which 2, 3 or 4 samples were analysed (Table 4). A mutation was found in 14. The detected allele frequencies had a mean average of 5.17%, with an SD of 2.36%. For each patient, the same mutation was identified in all tissues. The two patients without an identified somatic mutation, were each negative in 3 different samples.

**Table 4:** Patients with multiple samples analysed

Sequenced samples	Common code	Pathology	Mutation	VAF (%)	Average VAF (%)	SD VAF (%)
VA-10	VA-10	LM	E545G	10.01	8.28	2.45
VA-47.pKPT			E545G	6.55		
VA-1124.pKPT	VA-1124	CLOVES	E453K	13.51	8.64	6.89
VA-1133.pKPT			E453K	3.76		
VA-1126.pKPT	VA-1126	CLOVES	–	–	–	–
VA-1126.pRASO2			–	–		
VA-1126-T.pCMAVM			–	–		
VA-1158-T.pKPT	VA-1158-T	CLOVES	E545K	12.16	8.66	4.17
VA-1158-T-1.pTVPP			E545K	9.77		
VA-1158-T-2.pTVPP			E545K	4.05		
VA-230.pKPT	VA-230	LM	H1047L	2.52	2.50	0.04
VA-298.pKPT			H1047L	2.47		
VA-24.pKPT	VA-24	LM	–	–	–	–
VA-240.pKPT			–	–		
VA-986.pKPT			–	–		
VA-312.pKPT	VA-312	LM	E110delE	1.06	1.90	0.82
VA-339.pKPT			E110delE	1.95		
VA-403.pKPT			E110delE	2.70		
VA-364.pKPT	VA-364	LM	E542K	1.11	2.74	2.30
VA-421.pKPT			E542K	4.36		
VA-50.pKPT	VA-50	LM	E542K	4.95	6.56	2.28
VA-790.pKPT			E542K	8.17		
VA-528.pKPT	VA-528	LM	E545K	4.3	4.93	2.39
VA-714.pKPT			E545K	6.24		
VA-830.pKPT			E545K	7.32		
VA-913.pKPT			E545K	1.87		
VA-711.pKPT	VA-711	LM	H1047R	5.66	8.33	3.77
VA-829.pKPT			H1047R	10.99		
VA-756.pKPT	VA-756	LVM	E542K	8.25	7.07	1.67
VA-979.pKPT			E542K	5.89		
VA-1037.pKPT	VA-886	LM	Q546R	2.16	3.06	1.27
VA-886.pKPT			Q546R	3.96		
VA-868-T.pRASO2	VA-868	LM	E542K	2.11	3.78	2.36
VA-1245.pKPT			E542K	5.45		
VA-869-T.pRASO2	VA-869	LM	E545K	4.0	2.87	1.61
VA-1243.pKPT			E545K	1.73		
VA-1041.pKPT	VA-916	LM	H1047R	3.86	3.10	1.08
VA-916.pKPT			H1047R	2.33		

VAF, variant allele frequency; SD, standard deviation; mean of averages = 5.17%; mean of SDs = 2.36%

**Fig. 4:**
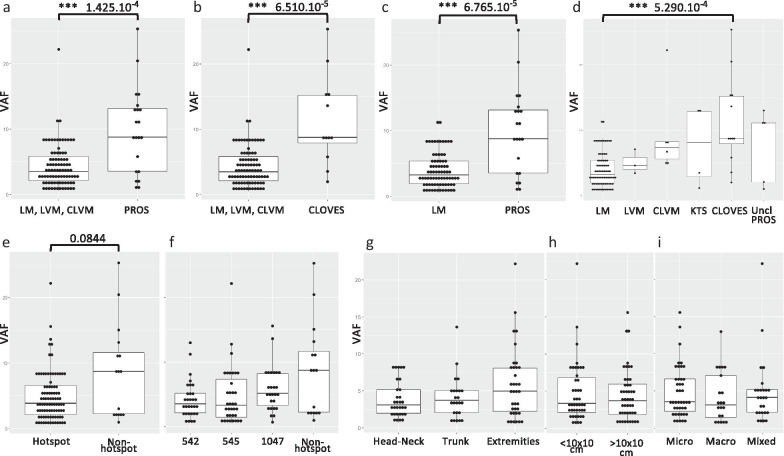
VAF distributions. **a** Highly significant difference in VAF between common and combined LMs (LM, LVM,CLM) versus syndromes (PROS), and **b** versus CLOVES. No statistically significant difference between common and combined LMs versus unclassified PROS or KTS, nor between LVM or CLVM versus syndromes. **c** Significant difference between common LM versus syndromes (PROS), and **d** versus CLOVES. There was no statistically significant difference between any other entities (Additional file 1: Table S1). **e, f**, no statistically significant difference between VAF and hostpot/non-hotspot or each of the hotspots individually (Additional file 1: Table S1). **g–i** no statistically significant differences between VAF and LM localization (**g**) (Additional file 1: Table S1), size (**h**) (Additional file 1: Table S1) or cystic structure (**i**) (Additional file 1: Table S1). ***p value < 0.001

Overall, VAF varied from 0.54 to 25.33% between different phenotypes (Table 1). There was a statistically highly significant difference in VAF between common and combined LMs versus syndromes (PROS) (*p* value = 1.425 × 10^–4^, Fig. 4a) and versus CLOVES (*p* value = 6.510 × 10^–5^, Fig. 4b), as well as between common LMs versus syndromes (PROS) (*p* value = 6.765 × 10^–5^, Fig. 4c) and versus CLOVES (*p* value = 5.290 × 10^–4^, Fig. 4d). There was no statistically significant difference between the other entities (Additional file 1: Table S1).

The VAF for hotspot mutations varied between 0.54 and 22.19% (n = 92, median: 3.88%, SD = 3.72), whereas for the non-hotspot mutations it varied between 1 and 25.33% (n = 16, median = 8.71%, SD = 7.27). There was no statistically significant difference between these two groups (*p* value = 0.0844) (Fig. 4e) or with individual hotspots (Fig. 4f, Additional file 1: Table S1). Finally, we analysed associations between VAF (Fig. 4g–i and Additional file 1: Table S1) or mutations (hotspot vs non-hotspot or presence vs absence) in regard to LM localization (Table 5), size (Table 6) or cystic structure (Table 7). There was no statistically significant difference identified in adjusted *p* values, except that a mutation is less often detected in small lesions (*p* value = 0.0129) (Table 6).

**Table 5:** Comparison of mutations versus localisation

Localisation	Mutation	Hotspot	Non-hotspot	No mutation
*Input data*				
Head & Neck	32	31	1	12
Trunk	25	23	2	11
Extremities	40	37	4	8

group_1	group_2	*p* value	Adjusted *p* value	
*Fisher's exact test Mutation/no mutation*				
Head & Neck	Trunk	0.8068	0.8068	
Head & Neck	Extremities	0.3118	0.4677	
Trunk	Extremities	0.1877	0.4677	
*Fisher's exact test Hotspot/non-hotspot*				
Head & Neck	Trunk	0.5762	0.8643	
Head & Neck	Extremities	0.3732	0.8643	
Trunk	Extremities	1	1

**Table 6:** Comparison of mutations versus lesional size

Size	Mutation	Hotspot	Non-hotspot	No mutation
*Input data*				
< 10 × 10 cm	46	45	1	23
> 10 × 10 cm	50	44	6	8
Unknown	1	1	0	0

group_1	group_2	*p* value	Significance	
*Fisher's exact test Mutation/no mutation*				
< 10 × 10 cm	> 10 × 10 cm	0.01285	*	

group_1	group_2	*p* value		
*Fisher's exact test Hotspot/non-hotspot*				
< 10 × 10 cm	> 10 × 10 cm	0.1134		

*p value < 0.05

**Table 7:** Comparison of mutations versus cystic structure

Cystic structure	Mutation	Hotspot	Non-hotspot	No mutation
*Input data*				
Microcystic	48	43	5	14
Macrocystic	21	21	0	13
Mixed	28	26	2	4
Unknown	11	2	9	4

group_1	group_2	*p* value	Adjusted *p* value	
*Fisher's exact test Mutation/no mutation*				
Microcystic	Macrocystic	0.1536	0.2304	
Microcystic	Mixed	0.2815	0.2815	
Macrocystic	Mixed	0.0241	0.0722	

group_1	group_2	*p* value	Adjusted *p* value	
*Fisher's exact test Hotspot/non-hotspot*				
Microcystic	Macrocystic	0.3132	0.7500	
Microcystic	Mixed	1.0000	1.0000	
Macrocystic	Mixed	0.5000	0.7500	

*Kruskal-Wallis chi-squared* = 5.906, df = 3, *p* value = 0.1163

## Discussion

We used deep sequencing and ddPCR to study a series of lymphatic malformations. Variant allele frequency ranged from 0.54 to 25.33%, suggesting that the most clonal surgical resections contained up to 50% of heterozygous mutant cells. In contrast, in the least clonal lesion detected, the frequency was as low as 1% of cells. VAF in common and combined LMs had a median of 3.50% (SD = 3.24); it was more than double in PROS samples (median = 8.78%, SD = 6.47). These are similar to the ranges previously reported [13, 14]. The higher mutant allele frequency detected in PROS patients suggests broader mosaicism.

We identified a statistically significant association between mutation types and phenotypes. The non-hotspot mutations had a higher frequency in CLOVES and unclassified PROS compared to common and combined LMs (Fig. 3). Similar has been noted for macrocephaly-capillary malformation (M-CM) in which PIK3CA mutations are more often non-hotspot mutations [15]. Moreover, M-CM patients tend to be more mosaic for the mutation, which is sometimes detectable in their blood [15]. The level of mosaicism in human tissues may reflect the potential pathogenicity (the strength of downstream gain-of-function effects) of a given mutation. This suggests that the non-hotspot mutations that are more frequently seen in widespread PROS (such as CLOVES and M-CM) may have weaker downstream effects and thus could occur earlier during fetal development, thereby affecting more extensive body parts. Interestingly, when KTS is defined as unilateral capillaro-lymphatico-venous malformation with hypertrophy, like in this study, it seems to have more hotspot mutations, like common and combined LMs which are usually localized.

The same amino acid substitutions are found in cancers resulting in activation of p110α [11]. As vascular anomalies do not transform into malignancy, the activating p110α mutations in ECs are not able to induce oncogenesis. Nevertheless, a study on 122 patients with CLOVES syndrome demonstrated a higher risk of developing a Wilms tumour (WT) [16]. This underscores the likely presence of CLOVES-associated mutations in cell types other than EC. Interestingly, two studies on patients with KTS reported that the risk of cancers in children and adults was not higher than in the general population [17, 18]. These reports fit with our notion that especially CLOVES patients tend to have non-hotspot mutations with higher allelic frequencies. A similar risk may also be true for other PROS patients that have a wider (bilateral) phenotype.

The implication of the same PIK3CA mutation in common LM and common VM reinforces the idea that the cell type in which mutations occur influences directly the pathology [9]. LMs would be due to somatic mutations in lymphatic endothelial cells (LEC), whereas VMs would be due to a mutation in blood endothelial cells (BEC). The cell-type specificity of mutations was underscored by the undetectable presence of the somatic PIK3CA mutation in our LM-derived fibroblasts and our CD-31 negative cells derived from a CLVM. Moreover, there was an increased detection rate of the tissular mutation (11%) in the LM-derived LECs (32%) (Table 2). This reinforces the idea that the somatic mutations occur in ECs or their precursors, determining the phenotype, and contrasts with CLOVES in which fibroblasts also harbour the mutation [19].

Intercellular signalling (mutant and wild-type) and cell–matrix interactions are also likely to play an essential role in the development of vascular malformations. This is highlighted by the single mutant cell type being at very low frequency (< 1%) in the lesion. Moreover, murine modelling demonstrated that the same somatic PIK3CA mutation activated in LECs can lead to macro- or microcystic lesions, depending on the time-point of induction of expression during development and growth [20]. Intra-uterine activation led to macrocystic LMs, whereas early post-natal induction led to microcystic LMs. This fits well with the concept of cell-type and time-dependent occurrence of somatic (hotspot)/mosaic (non-hotspot) mutations explaining variability in phenotype [21].

Our results indicate that PI3K-pathway inhibition could work in at least 75.5% of patients with a lymphatic malformation. In the three published prospective clinical trials using Rapamycin, an mTOR inhibitor, on patients affected by various vascular malformations, response rates were high [22–25]. In one study, all 6 patients had a somatic mutation activating PI3K signalling and the response rate was 100%. In the other two studies, somatic genotyping had been performed only for a minority of the patients, yet > 85% had at least a partial response after 12 months of treatment. A PIK3CA inhibitor, BYL719 or Alpelisib, was tested on 19 patient with PROS, also showing good outcomes [26]. Similarly, the Pan-ALK inhibitor ARQ 092 (Miransertib) showed promising results on a cohort of 6 patients with PIK3CA-Related Overgrowth Syndrome [27]. These results demonstrate the positive impact of repurposing oncology drugs to patients suffering from benign vascular anomalies with a proven or likely somatic mutation that activates PI3K.

Genotyping is likely crucial in future clinical trials to increase efficacy. The high frequency of mutations identified in this study (75.5%) suggests that LMs are mostly caused by a somatic *PIK3CA* mutation. However, one fourth does not seem to have a *PIK3CA* mutation. This could be due to low representativeness of mutant ECs in the studied tissue sample. Analysis of multiple lesional samples may increase rate of molecular diagnosis [14]. Yet, we confirmed negativity in additional samples when available (n = 2 patients), and in the other 14 patients that were sequenced two, three, or four times, the mutation was found in all samples, albeit with variability in VAFs (Table 4).

## Conclusion

In conclusion, our systematic data on a large cohort of patients with lymphatic malformations demonstrate that 75.5% of LMs, whether common, combined, or syndromic, are caused by somatic activating PIK3CA mutations. There is a statistically significant difference in mutation types between common and combined LM versus syndromic LMs (CLOVES and unclassified PROS), suggesting differential effects of the mutations. Non-hotspot mutations need to be looked for especially in the more wide-spread PROS phenotypes. Based on these and earlier data, repurposing of PI3K signalling pathway inhibitors for the treatment of LMs, whether isolated, combined or syndromic, have a sound epidemiological and pathophysiological basis.

## Methods

### Aim

Identification of prevalence of PIK3CA mutations and genotype–phenotype correlations in pure and combined lymphatic malformations.

### Design

Tissues were collected from the leftover of programmed surgeries and snap-frozen in liquid nitrogen. Clinical data in regard to the phenotype (Table 1), and localization (head & neck, trunk and extremities), size (smaller or larger than 10 × 10 cm) and cystic structure (micro, macro, mixed) of the lesions were collected (Table 8). Nine of the patients have been included in earlier reports: one KTS as patient #5 in (9) and the associated E545K mutation in (11); five common LMs with H1047R with histology as patients #1–5 in (22), and two LMs (patients #2 and #6) as well as one KTS (patient #18) in a clinical trial (25).

**Table 8:** Summary of samples per cystic structure, size and localisation

Cystic structure	Mutation	Hotspot				Non-hotspot	No mutation	Total	% mutation
Size*			542K	545K	1047				
**Microcystic**	**48**	***43***	14	15	14	***5***	**14**	**62**	**77%**
< 10 × 10 cm	20 (7/4/9)	19 (7/3/9)	10	4	5	1 (0/1/0)	9 (3/3/3)	29	69%
> 10 × 10 cm	27 (6/10/11)	23 (5/9/9)	4	11	8	4 (1/1/2)	5 (0/2/3)	32	84%
Unknown	1 (0/1/0)	1 (0/1/0)	0	0	1	0	0	1	100%
**Macrocystic**	**21**	***21***	9	10	2	***0***	**13**	**34**	**62%**
< 10 × 10 cm	16 (7/2/7)	16 (7/2/7)	6	9	1	0	12 (5/6/1)	28	57%
> 10 × 10 cm	5 (2/1/2)	5 (2/1/2)	3	1	1	0	1 (1/0/0)	6	83%
Unknown	0	0	0	0	0	0	0	0	–
**Mixed**	**28**	***26***	8	9	9	***2***	**4**	**32**	**88%**
< 10 × 10 cm	10 (4/3/3)	10 (4/3/3)	4	4	2	0	2 (2/0/0)	12	83%
> 10 × 10 cm	18 (6/4/8)	16 (6/4/6)	4	5	7	2 (0/0/2)	2 (1/0/1)	20	90%
Unknown	0	0	0	0	0	0	0	0	–
**Unknown**	**11**	***2***	0	1	1	***9***	**4**	**15**	**73%**
***Total***	***108***	***92***	***31***	***35***	***26***	***16***	***35***	***143***	***76%***

*Numbers per localisation given between parenthesis: (Head&Neck/Trunk/Extremities). See Tables 6 and 7 for globalized numbers per size and localization. Mutation numbers are subdivided in Hotspot and Non-hotspot, with Hotspot numbers further subdivided per position

Targeted next-generation sequencing (NGS) or ddPCR to screen for somatic PIK3CA mutations on DNA extracted from resected lesional tissue or lymphatic endothelial cells (LECs) isolated from lesions.

### DNA extraction

DNA extraction from frozen tissues as previously described [9]. DNA was quantified using NanoDrop 8000 (Thermo Fisher Scientific) and Qubit 2.0 (Thermo Fisher Scientific).

### Isolation and culturing of primary endothelial cells (EC) and primary lymphatic ECs

Single-cell solutions were obtained from CLVM and LM tissues by digesting with 0.04% dispase (Gibco), 0.25% collagenase II (Roche) and 0.01% DNAse I (Roche) for 1 h at 37 °C. Separated cells (= mixed cells) were seeded on fibronectin-coated flasks (2 μg/cm^2^) (Millipore) and grown in ECGM2 (Bio-Connect life sciences) for CLVM isolated cells and ECGM-MV2 (Bio-Connect life sciences) for LM isolated cells, both supplemented with penicillin–streptomycin. When mixed cells reached a confluency of 80%, they were detached using Accutase (Sigma). Mixed CLVM cells were sorted for CD31-positive cells using Anti-CD31 MicroBeads (Miltenyi). To obtain LECs, we performed a fibroblast depletion using Anti-fibroblast MicroBeads (Miltenyi) as well as CD34-positive blood EC depletion using Anti-CD34 MicroBeads (Miltenyi), followed by a CD31-positive selection using Anti-CD31 MicroBeads. All different selected cells were plated on fibronectin-coated flasks (2 μg/cm^2^). Cell morphology was confirmed using a bright field microscope (Zeiss).

### PIK3CA sequencing

We designed an Ion AmpliSeq panel for targeted sequencing of the 21 coding exons of *PIK3CA* and ten nucleotides of all flanking introns (NM_006218.2) (http://www.ampliseq.com). Theoretical horizontal coverage was 96.75%, with 16 bp in the 5’UTR (exon 1) and 32 bp in exon 20 not covered. The panel consisted of 2 pools of primers for multiplexed PCR-amplification with Ion Ampliseq Library kit, and sequencing on an Ion Personal Genome Machine (PGM) or an ion Proton (Thermo Fisher Scientific). Reads were aligned to the human reference sequence hg19, using the Torrent Suite Server. Bam files were imported into Highlander software package (https://sites.uclouvain.be/highlander/) for analysis. We selected variants with at least 5 mutant reads representing at minimum 1% of all alleles by interrogating all positions reported with at least 4 changes in the COSMIC database (https://cancer.sanger.ac.uk/cosmic). Samples needed to have an average coverage above 500 × to be considered not to contain a mutation. Mutations with a VAF below 1% but confirmed by ddPCR when DNA was available, were considered to contain a mutation.

### Digital droplet PCR (ddPCR)

Digital droplet PCR (ddPCR) was used to study four known PIK3CA hotspot mutations: *c.1624G* > *A* (p.E542K), *c.1633G* > *A* (p.E545K), *c.3140A* > *T* (p.H1047L) and *c.3140A* > *G* (p.H1047R) (NM_006218.2). Probes were designed by Bio-Rad (Bio-Rad Laboratories). The ddPCR experiments were performed, as described by Hindson et al*.* [28]. DNA input was 30 ng. At most, 93 samples were run in parallel. For the analysis, we used QuantaSoft software (Version 1.7). Samples had to have at least 5 mutant single positive droplets (SPD) among a minimum of 10,000 droplets to be considered mutated. Nine patients with detection of mutation by ddPCR could not be tested by NGS. Their variant allele frequencies ranged from 1.6 to 15.6%.

### Statistics

Data were analyzed to detect differences between groups using Fisher’s exact test for two categorical variables, Pairwise Wilcoxon’s rank-sum test for group comparisons of continuous variables, and the Kruskal–Wallis test for > 2 group comparisons of continuous variables. Nonparametric tests were used due to small sample size and non-normal distributions (tested with Shapiro test). Significance of *p* values: *p ≤ 0.05, **p ≤ 0.01, and ***p ≤ 0.001. In order to control type I errors, multiple testing corrections were performed using Benjamini–Hochberg procedure. All analyses were performed using R and graphs were generated with ggplot2. Descriptive statistics were computed using groupedstats R package. VAF values for statistics were based on NGS results (average VAF values for patients with multiple sequencing, Table 4) or ddPCR (for 10 samples tested only by ddPCR).

## Supplementary Information

**Additional file 1.** Statistical comparisons of data shown in Fig. 4 d,f, g, h, i.

## Data Availability

The datasets used and/or analysed during the current study are available from the corresponding author on reasonable request.
