# Supplementary material for: Non-hotspot PIK3CA mutations are more frequent in CLOVES than in common or combined lymphatic malformations
Source: Orphanet J Rare Dis. 2021 Jun 10;16:267. doi: 10.1186/s13023-021-01898-y (PMC8194016; doi:10.1186/s13023-021-01898-y)
Supplement: Supplementary file 1 — Additional file 1. Statistical comparisons of data shown in Fig. 4 d,f, g, h, i. [file 13023_2021_1898_MOESM1_ESM.docx]

**Supplementary Table 1. Statistical results**

| **Wilcoxon analysis of VAF per pathology** (*see also Figure 4d*) | | | | | |
| --- | --- | --- | --- | --- | --- |
| **Test** | **CLOVES** | **CLVM** | **KT** | **LM** | **LVM** |
| **CLVM** | 0,51856599 |  |  |  |  |
| **KT** | 0,51856599 | 0,90659341 |  |  |  |
| **LM** | 0,00052877 | 0,05755604 | 0,51856599 |  |  |
| **LVM** | 0,48319976 | 0,51856599 | 0,91836735 | 0,51856599 |  |
| **PROS** | 0,64336848 | 1 | 0,90659341 | 0,51856599 | 0,90659341 |
| **Wilcoxon analysis of VAF per Mutation** (*see also Figure 4f*) | | | | | |
| **Test** | **1047** | **542** | **545** |  |  |
| **542** | 0,13440858 |  |  |  |  |
| **545** | 0,13440858 | 0,93344262 |  |  |  |
| **nhs** | 0,47987759 | 0,14160238 | 0,13440858 |  |  |
| **Wilcoxon analysis of VAF per localisation** (*see also Figure 4g*) | | | | | |
| **Test** | **Trunk** | **Head & Neck** |  |  |  |
| **Head & Neck** | 0,76832923 |  |  |  |  |
| **Extremities** | 0,38661312 | 0,38661312 |  |  |  |
| **Wilcoxon analysis of VAF per size** (*see also Figure 4h*) | | | | | |
| **Test** | **<10x10cm** |  |  |  |  |
| **>10x10cm** | 0,99033958 |  |  |  |  |
| **Wilcoxon analysis of VAF per cystic structure** (*see also Figure 4i*) | | | | | |
| **Test** | **Macrocystic** | **Microcystic** |  |  |  |
| Microcystic | 0,5125047 |  |  |  |  |
| Mixed cystic | 0,65127276 | 0,65127276 |  |  |  |
